# Case report: One human *Streptococcus suis* infection in Shandong Province, China

**DOI:** 10.1097/MD.0000000000033491

**Published:** 2023-04-07

**Authors:** Yuwei Liu, Zengqiang Kou, Xin Wang, Shuyu Chen, Renpeng Li, Qiang Wang

**Affiliations:** a College of Public Health, Weifang Medical University, Weifang, P.R. China; b Shandong Center for Disease Control and Prevention, Weifang, P.R. China; c Department of Epidemiology, Weifang Medical University, Weifang, P.R. China.

**Keywords:** burden, case report, cerebrospinal fluid culture, pigs, *Streptococcus suis*

## Abstract

*Streptococcus suis* is an emerging zoonotic pathogen. Human infections with *S suis* have been identified in Europe, North America, South America, Oceania, Africa and Asia. As the most common clinical symptom of human *S suis*, meningitis develops in 50% to 60% of infected patients, and approximately 60% of the patients with meningitis symptoms have neurologic sequelae. The cost of infection with *S suis* imposes a tremendous burden on patients’ families.

**Case presentation:**

A 56-year-old woman was infected with *S suis*. The patient reared pigs in her backyard. At admission, her blood examination showed a leukocyte count of 27.28 × 109/L with 94.20% neutrophils. Cerebrospinal fluid was cloudy with a leukocyte count of 2700 × 106/L. Cerebrospinal fluid cultures revealed gram-positive cocci identified as *S suis* type II. Ceftriaxone was then administered.

**Conclusion:**

Human infections with *S suis* highlights the need for health education, prevention and surveillance it.

## 1. Introduction

Since the infection of human with *Streptococcus suis* was first identified in 1968,^[1]^ *S suis* has become an emerging zoonotic pathogen.^[2]^ Human infections with *S suis* have been identified in Europe, North America, South America, Oceania, Africa and Asia.^[3–5]^ As the most common clinical symptom of human *S suis*, meningitis develops in 50% to 60% of infected patients, and approximately 60% of the patients with meningitis symptoms have neurologic sequelae.^[5,6]^ In addition, hearing disorder with or without vestibular dysfunction appears in more than 50% of the patients with meningitis symptoms.^[5,7]^ The fatality rate of human *S suis* infections is approximately 12% worldwide but more than 18% in China.^[8–10]^ The cost of infection with *S suis* imposes a tremendous burden on patients’ families.^[11]^ Experiencing traumatic injuries while interacting with pigs infected by *S suis*, rearing pigs infected by *S suis*, slaughtering pigs infected by *S suis*, preparing pork and consuming undercooked pork may increase the risk of *S suis* infection.^[2,5]^ Although human *S suis* infection is sporadic in most areas of the world,^[4]^ it is an increasing public health threat in China due to the tradition of consuming pork and rearing pigs in backyards.^[6,12,13]^ Thus, the public health threat resulting from human *S suis* infections emphasizes the importance of epidemiological surveillance of *S suis* in humans in China. Here, we report 1 case of human *S suis* infection in Shandong Province, China.

## 2. Case presentation

A 56-year-old previously healthy woman presented with a 1-day history of fever (38.7°C), headache, nausea and vomiting. She reared pigs in her backyard. She was first admitted to a local community hospital on January 18, 2018. Computed tomography of the head revealed no abnormalities. Symptomatic antipyretic treatment was administered. To achieve a definitive diagnosis and receive further treatment, she was transferred to the Affiliated Hospital of Jining Medical University with a diagnosis of infection of the central nervous system on January 19, 2018. At admission, the patient had a body temperature of 37.7°C and a blood pressure of 140/99 mm Hg. Blood examinations showed a leukocyte count of 27.28 × 10^9^/L with 94.20% neutrophils. Cerebrospinal fluid was noted to be cloudy with a leukocyte count of 2700 × 10^6^/L, and cerebrospinal fluid biochemical analysis showed a protein level of 2.82 g/L. Magnetic resonance and contrast-enhanced magnetic resonance scans showed abnormally high signals in the bilateral sulcus. Pandy’s test was positive (1+). Cerebrospinal fluid culture grew gram-positive cocci that were identified as *S suis* type II. Antibiotic susceptibility testing showed that the pathogen was susceptible to ceftriaxone, vancomycin, levofloxacin, penicillin and ampicillin. Then, treatment with ceftriaxone was initiated. On February 11, 2018, cerebrospinal fluid biochemical analysis showed a protein level of 0.45 g/L and a leukocyte count of 38 × 10^6^/L. In addition, Pandy’s test was negative. The patient was discharged voluntarily form the hospital after recovery and was prescribed antibiotics.

This study was approved by the Ethics Review Committee of Weifang Medical University, and the participant provided informed consent.

## 3. Discussion

Occupational exposure to swine is an independent risk factor for *S suis* infection.^[4,8]^ In addition to contact transmission,^[8]^ airborne transmission of *S suis* has been identified.^[14]^ Although it was not known whether the patient’s pigs were infected by *S suis*, it may be supposed that the patient in this article was infected by *S suis* though pigs based on her history of rearing pigs and cerebrospinal fluid culture results. Furthermore, the preparation and consumption of pork, especially if skin injuries occur while handling pigs and pork, may also lead to *S suis* infection.^[4,8]^ Thus, occupational protection is necessary for people who work on pig farms and in pig slaughterhouses.

Consistent with previous reports, the patient’s leukocyte counts in the blood and cerebrospinal fluid in this article were higher than normal.^[2,3,5,7]^ In line with previous studies, the patient in this article was diagnosed definitely based on cerebrospinal fluid culture.^[5,7]^ In addition, blood culture is also an effective method of definitely diagnosing *S suis* infection.^[2,3]^ If the patient has a history of contact with pigs or pork, high leukocyte counts in the blood and cerebrospinal fluid may be treated as signs of *S suis* infection. In addition, blood culture and cerebrospinal fluid culture can be used to definitely diagnose *S suis* infection.^[8]^ Meningitis, which was observed in the patient in this report, is the most common clinical syndrome caused by human *S suis* infection.^[4,8]^ In addition, sepsis, arthritis, endocarditis and endophthalmitis are clinical syndromes resulting from human *S suis* infection.^[4,8]^ Broad-spectrum antimicrobials have been suggested as treatment for human *S suis* infection.^[4]^ However, the antibiotic susceptibility testing results of human infections with *S suis* differed from each other in previous studies.^[2,7]^ Moreover, antibiotic resistance has been found strains of *S suis* isolated from humans.^[4,6]^ Thus, antibiotic susceptibility testing is necessary for each patient infected with *S suis* to enable the provision of precise treatment.

Although human *S suis* infection is generally sporadic in China, its financial burden is disastrous for the patients’ families. The cost per human *S suis* infection is greater than the annual per capita family income.^[11]^ Patients with *S suis* infections are often the main financial supporters of their families.^[11]^ Human *S suis* infections may result in neurologic sequelae, hearing loss and even death,^[5,6,8–10]^ which may lead to the partial or complete loss of the family income. Compounding this issue is the fact that few people are aware of human *S suis* infections or the need to take preventive measures. This situation may contribute to the occurrence of *S suis* infections. Therefore, surveillance and health education about human *S suis* infections are still needed.

## 4. Conclusion

In this article, we report one case of human *S suis* infection. Although human *S suis* infection is sporadic in China, the burdens imposed on patients’ families and society highlight the need for health education, prevention and surveillance effort.

## Author contributions

**Data curation:** Yuwei Liu, Xin Wang, Shuyu Chen, Renpeng Li.

**Investigation:** Yuwei Liu, Renpeng Li, Zengqiang Kou, Qiang Wang.

**Writing – original draft:** Yuwei Liu, Qiang Wang.

**Software:** Xin Wang, Shuyu Chen, Zengqiang Kou.

**Resources:** Zengqiang Kou.
